# Assessing the Quality of Online Health Information About Breast Cancer from Chinese Language Websites: Quality Assessment Survey

**DOI:** 10.2196/25783

**Published:** 2021-11-18

**Authors:** Weiwei Sun, Aijing Luo, Zhiwei Bian, Bin Zhao, Peng Liu, Kai Wang, Yuwen Liu, Wenzhao Xie, Fuzhi Wang

**Affiliations:** 1 School of Health Management Bengbu Medical College Bengbu China; 2 The Second Xiangya Hospital Central South University Changsha China; 3 Key Laboratory of Medical Information Research College of Hunan Province Central South University Changsha China; 4 The Third Xiangya Hospital Central South University Cahngsha China

**Keywords:** online health information, breast cancer, Chinese language websites, quantitative evaluations

## Abstract

**Background:**

In China, the internet has become one of the most important ways to obtain information about breast cancer. However, quantitative evaluations of the quality of Chinese health websites and the breast cancer treatment information they publish are lacking.

**Objective:**

This study aimed to evaluate the quality of Chinese breast cancer websites and the value, suitability, and accuracy of the breast cancer treatment information they publish.

**Methods:**

Chinese breast cancer health websites were searched and manually screened according to their Alexa and Baidu search engine rankings. For each website included in the survey, which was conducted on April 8, 2019, the three most recently published papers on the website that met the inclusion criteria were included for evaluation. Three raters assessed all materials using the LIDA, DISCERN, and Suitability Assessment of Materials (SAM) tools and the Michigan Checklist. Data analysis was completed with the Statistical Package for Social Sciences (SPSS) version 20.0 and Microsoft Excel 2010.

**Results:**

This survey included 20 Chinese breast cancer websites and 60 papers on breast cancer treatment. The LIDA tool was used to evaluate the quality of the 20 websites. The LIDA’s scores of the websites (mean=54.85, SD 3.498; total possible score=81) were low. In terms of the layout, color scheme, search facility, browsing facility, integration of nontextual media, submission of comments, declaration of objectives, content production method, and robust method, more than half of the websites scored 0 (never) or 1 (sometimes). For the online breast cancer treatment papers, the scores were generally low. Regarding suitability, 32 (53.33%) papers were evaluated as presenting unsuitable material. Regarding accuracy, the problems were that the papers were largely not original (44/60, 73%) and lacked references (46/60, 77%).

**Conclusions:**

The quality of Chinese breast cancer websites is poor. The color schemes, text settings, user comment submission functions, and language designs should be improved. The quality of Chinese online breast cancer treatment information is poor; the information has little value to users, and pictorial information is scarcely used. The online breast cancer treatment information is accurate but lacks originality and references. Website developers, governments, and medical professionals should play a full role in the design of health websites, the regulation of online health information, and the use of online health information.

## Introduction

Breast cancer is a malignant tumor of the breast epithelium. Since 1980, the incidence of breast cancer has been increasing worldwide. The age-standardized mortality rate based on the world standard population was 182.6 per million in 2018, and breast cancer is the leading cause of death due to cancer in women (15.0%) [1]. In the past decade, the incidence of breast cancer in China has also been rapidly growing, with a prevalence of 1%-2%, higher than that in other countries [2]. According to the latest national cancer statistics released by the National Cancer Centre of China in January 2019, breast cancer is the fourth-leading cause of death due to cancer among women in China and is one of the malignant tumors threatening the health of Chinese women [3].

For cancer-related information, the majority of the public is more likely to search the internet first. Because cancer is a major disease that is associated with strong privacy and sensitivity, breast cancer patients tend to first seek relevant health information from the internet to deepen their understanding of the disease and assist them in making decisions on health behavior [4]. The process of searching for health information about breast cancer is influenced by subjective and objective factors. The subjective factors include the users' information literacy level. Some research results have shown that the educational level of internet users [5], their attitude toward online health information [6], and their ability to acquire [7] and evaluate [8] online health information have positive effects on their rational utilization of this type of information. The objective factors mainly include the quality of online health information.

In Europe and North America, there is much research on online cancer health information [9,10]. The quality assessment of online health information about cancer has attracted extensive attention from scholars worldwide. Garfinkle [11] evaluated the readability, quality, and accuracy of online health information for patients with low anterior resection syndrome following surgery for rectal cancer and found that online health information is lacking and too complex for patients to understand. Another study [12] assessed the availability and quality of information about female oncofertility on the websites of (inter)national oncology, fertility, and oncofertility organizations and suggested that the availability and quality of online health information be improved and that high-quality resources be recommended by physicians. The quality of online health information is a complex concept involving more than 20 dimensions, as perceived by consumers. The most widely reported criteria used by consumers were trustworthiness, expertise, and objectivity, and the most widely reported indicators were website owner/sponsor, consensus among multiple sources, the characteristics of writing and language, advertisements, content authorship, and interface design [13].

In China, according to the 43rd China Internet Network Development Statistics report released by the China Internet Network Information Centre, as of December 2018, China had 829 million internet users [14]. Online health information services have become an important way for people to obtain health information [15]. The quality of health information service platforms is affected by the quality of the websites and the information they publish [16]. However, due to the imbalance between the rapid development of informatization and the regulation of online information, the quality of online health information service websites and their information has been uneven [17]. According to the results of a recent study [18], Baidu is the most popular online information source for breast cancer; however, more than half (55.1%) of those surveyed were dissatisfied with the online information. To date, China's domestic research on online health information has focused on discussing online health information evaluation indexes [19] and evaluation tools [20] and on theoretical research on online health information service platforms [21]. However, there is still a lack of in-depth research on the quantitative assessment of health information websites and the information they publish.

Currently, there are many tools for online information quality evaluation. Some evaluation tools are highly targeted. For example, the Suitability Assessment of Materials (SAM) tool can be used to evaluate the applicability of information [22]. The Simplified Measure of Gobbledygook (SMOG) tool can be used to evaluate the readability of information [23], while the Health on the Net Foundation Code of Conduct (HONcode) has proposed a special code of ethics for the release of online health information for health websites [24]. Other tools focus on evaluating the quality of online health information from multiple dimensions; for example, the Michigan Checklist includes an evaluation of online health information quality and website design [25]. In addition to assessing the information quality of online treatment schemes, the DISCERN tool uses several items to evaluate the reliability of websites and has been used to evaluate the readability, suitability, and quality of online health information [22] In this study, we carefully examined relevant online health information quality evaluation tools and assessed the effectiveness of these tools and the independence between subdimensions, finding that some subdimensions of these evaluation tools are repeated. To evaluate the quality of online breast cancer health information as comprehensively as possible and to avoid duplication of the subdimensions of the evaluation tools, we chose to conduct our study based on the two dimensions of website quality (usability and reliability) and three dimensions of information quality (value, suitability, and accuracy).

The purpose of this study was to evaluate the quality of Chinese health breast cancer websites and to evaluate the quality of online breast cancer treatment information in terms of value, suitability, and accuracy. This study aims to provide support for breast cancer patients and caregivers to make effective use of online health information services and to make reasonable health decisions by analyzing the quality of and the problems with online health information in China.

## Methods

### Sample

The evaluation of the quality of online health information about breast cancer was divided into two parts: First, the quality of Chinese websites that publish breast cancer health information was evaluated; second, the quality of Chinese online papers on breast cancer treatment was evaluated. The quality of websites mainly depends on their functionality. The quality of papers mainly depends on the health-related content.

The initial screening of Chinese breast cancer websites was completed in two steps. The first step consisted of selecting the top 100 websites as research samples based on the results of Chinese medical and health websites provided by Webmaster’s House and the Alexa ranking. The second step consisted of using the Baidu search engine to select the results of the first 20 pages from the list of search results, with “breast cancer” or “breast tumor” used as the search keyword. In all, 38 breast cancer–related health websites were manually screened. ChinaZ^TM^ is the most well-known basic web service provider in China, providing users with Alexa ranking queries, website traffic queries, and other services on Chinese websites. Alexa has the largest number of Uniform Resource Locators (URLs) and detailed ranking information [26]. Alexa China provides free official data queries of Chinese website rankings, which can reflect the traffic and popularity of a website to some extent [27]. Baidu is the most visited Chinese search engine in the world [28,29]. Based on the search results of these two platforms and after eliminating 26 duplicate websites, 112 websites were included in the research sample pool of this study. The inclusion criteria for the health websites were as follows: (1) the websites were Chinese websites, (2) the information released by the websites was obviously relevant to breast cancer health, (3) the websites were not intended to sell merchandise, and (4) the websites were not official hospital websites. We excluded hospital websites because official websites provide basic information about the hospital, such as an introduction to the hospital and departments. Moreover, there is little detailed health information about breast cancer on hospital websites. Based on the inclusion criteria, 20 breast cancer health websites were finally included in the survey.

The inclusion criteria for Chinese online papers on breast cancer were as follows: (1) the papers were written in Chinese, (2) the papers were related to breast cancer treatment information, (3) the papers were not for advertising, and (4) the papers included text and pictures. According to the date of publication of the papers, three recently published papers on each website that met the inclusion criteria were selected as samples to evaluate the quality of online treatment information about breast cancer.

The samples were was collected on April 8, 2019. The sample collection process is shown in Multimedia Appendix 1.

### Tools

#### Quality Assessment Tool for Websites

LIDA was used to evaluate the usability and reliability of health websites on breast cancer. This tool was developed by Minervation, a British consulting company in the health care field, in 2007 and was designed for professionals to evaluate all aspects of health websites, focusing on the degree of recognition of health websites by professionals [30]. The evaluation of usability included four dimensions: clarity, consistency, functionality, and engageability. The evaluation of reliability included three dimensions: currency, conflicts of interest, and content production. In this study, a total of 27 items were used to evaluate the websites. Each question was scored on a scale of 0 to 3, where 0 indicated never, 1 indicated sometimes, 2 indicated mostly, and 3 indicated always.

#### Quality Assessment Tool for Papers

The quality of the papers was assessed based on three key parameters: value, suitability, and accuracy.

#### Value Assessment Tool

The value of online papers on breast cancer was assessed using a 7-item scale selected from the DISCERN tool. DISCERN is a tool for judging the quality of written consumer health information about treatment choices; it was developed by the British Library in 1999 [31]. DISCERN consists of a total of 16 questions, and it was the first tool in the world for evaluating the information quality of health websites. It includes three dimensions: the evaluation of websites, the evaluation of the value of therapeutic papers, and overall evaluation. DISCERN is a validated tool that has adequate internal consistency (α=.78) and satisfactory interrater reliability [32]. To assess the value of papers on breast cancer treatment, we selected only the second dimension of the DISCERN scale. It includes 7 questions, each rated on a 5-point Likert scale ranging from 1=no (ie, the criterion is not fulfilled by the publication) to 5=yes (ie, the criterion is fulfilled by the publication).

#### Suitability Assessment Tool

The SAM tool was used to evaluate the suitability of online health information about breast cancer. SAM, developed and designed by Doak, is an objective assessment tool for evaluating the availability and reliability of health materials [33]. SAM includes 6 dimensions: content (4 items), literacy demand (5 items), graphics (5 items), layout and typography (3 items), learning stimulation and motivation (3 items), and cultural appropriateness (2 items). Each item is scored on a scale of 0 to 2 points, where 0 indicates not suitable, 1 indicates adequate, and 2 indicates superior. In this study, considering that there was no front cover with online health information, we removed one item (cover graphic) that did not apply. The higher the final score of a paper is, the better its suitability.

#### Accuracy Assessment Tool

Six items in the Michigan Checklist were selected to evaluate the accuracy of the papers. The Michigan Checklist was created by the University of Michigan in 1999, and it focuses on evaluating health websites and their content. The scale included two aspects: content and usability. Because this study mainly evaluated the accuracy of papers published on breast cancer websites, the content of the scale (items 18-23) was selected to evaluate the accuracy of the papers:

- #18. Are sources cited or credited?
- #19. Is a bibliography or resource list available?
- #20. Can you identify errors or significant omissions in information presented?
- #21. Are opinions or misleading/biased information presented as fact?
- #22. Does information presented as factual appear to be accurate to the best of your knowledge?
- #23. Is there an identifiable conflict of interest?

### Rating Process

The evaluation was performed by three assessors. Assessor 1 (author SWW) holds a master’s degree in medical informatics and has 7 years of experience in medical information analysis and research. Assessor 2 (author WFZ) holds a master's degree in computer science and a doctorate in social medicine and has 8 years of experience in computer software development. Assessor 3 (author BZW) holds a doctor of medicine degree and a clinical physician qualification certificate. Two websites (six papers) were used for experimental evaluation. Before the test, the three researchers (assessors) carefully read the scales and usage instructions of the four assessment tools (the LIDA, DISCERN, and SAM tools and the Michigan Checklist) to understand the purpose and significance of the evaluation items. The evaluation was divided into two steps. First, assessors 1 and 2 used LIDA to evaluate the quality of the websites. Then, assessors 1 and 3 used the other three scales to evaluate the quality of the selected papers. To ensure the consistency of the evaluation results, the subdimension was adopted as the evaluation unit; that is, the N-th+1 dimension was evaluated after the evaluation of the N-th dimension of all samples was completed. The evaluation process adopted a parallel mode. Two assessors independently evaluated a given sample simultaneously. In the case of diverging evaluation results, the final results were determined through real-time negotiation.

### Ethical Approval

Ethical approval for the study was obtained from the Medical Ethics Committee of the BengBu Medical College (BBMC; reference no. 2017054).

### Statistical Analysis

Analysis was conducted using the Statistical Package for Social Sciences (SPSS) version 20.0 (SPSS Inc., Chicago, IL, USA) and Microsoft Excel 2010 (Microsoft Inc., Washington DC, USA). All values are expressed as the mean ± SD.

## Results

### Characteristics of the Breast Cancer Websites

The characteristics of the breast cancer websites are shown in Table 1. All 20 websites had internet content provider (ICP) registration numbers. Of the 20 websites, 17 (85%) were corporate websites and 3 (15%) were personal websites. There were 3 (15%) websites with Baidu weights of 7-9, 10 (50%) websites with Baidu weights of 4-6, and 7 (35%) websites with Baidu weights of less than 3. Regarding the number of years since website registration, there were 4 (20%) websites that had been registered for more than 15 years, 8 (40%) websites that had been registered for 5-10 years, and only 1 (5%) website that had been registered for less than 5 years. In terms of regional distribution, 17 (85%) websites were registered in eastern China, 1 (5%) was registered in central China, and 2 (10%) were registered in western China.

**Table 1:** Characteristics of the breast cancer websites (N=20).

Characteristic	Group	n (%)
Nature	Enterprise	17 (85)
	Personal	3 (15)
Global ranking	Less than 10,000	4 (20)
	10,000-30,000	5 (25)
	30,000-60,000	2 (10)
	More than 60,000	6 (30)
	—^a^	3 (15)
Traffic ranking	10,000	4 (20)
	10,000-30,000	5 (25)
	30,000-60,000	3 (15)
	More 60,000	5 (25)
	—	3 (15)
Week of Alexa ranking	5000	2 (10)
	5000-10,000	2 (10)
	10,000-30,000	6 (30)
	More than 30,000	4 (20)
	—	6 (30)
ICP^b^ certified	Yes	20 (100)
	No	0 (0)
Baidu weight^c^	7-9	3 (15)
	4-6	10 (50)
	Less than 3	7 (35)
Years since registration	More than 15	4 (20)
	10-15	8 (40)
	5-10	7 (35)
	Less than 5	1 (5)
Region	Eastern part	17 (85)
	Central part	1 (5)
	Western part	2 (10)

^a^—: not available.

^b^ICP: internet content provider. ICPs are telecom operators providing comprehensive internet information services and value-added services to a vast number of users. The required certificate is the ICP certificate. Profit-making websites must handle ICP certificates; otherwise, they are illegal businesses.

^c^Baidu weight: Baidu weights are evaluated data that are used to estimate search engine traffic by the webmaster tool through an analysis of the ranking of a website’s keywords. The evaluated data are divided into 0-9 for a total of 10 grades. Baidu weights are related to the number of keywords and traffic. The more keywords there are, the higher the weight of accumulation. The higher the keyword flow is, the higher the cumulative weight will be.

### Quality of Breast Cancer Websites

We used LIDA to evaluate the quality (usability and reliability) of the 20 breast cancer websites. The evaluation results are shown in Table 2. The evaluation results showed that the overall score of the quality evaluation of the breast cancer websites was 54.85±3.498 (81 points). With regard to the layout, color scheme, search facility, browsing facility, integration of nontextual media, submission of comments, declaration of objectives, content production method, and robust method, the scores of the websites were low. The results showed that the quality of the websites needs to be improved.

**Table 2:** Descriptive statistics of LIDA items.

Item		Score=0	Score=1	Score=2	Score=3	Mean (SD)
**2.1 Clarity, n (%)**						
	2.1.1 User scope	0 (0)	1 (5)	19 (95)	0 (0)	1.950 (0.224)
	2.1.2 Knowledge level	0 (0)	3 (15)	16 (80)	1 (5)	1.900 (0.447)
	2.1.3 Layout	0 (0)	13 (65)	6 (30)	1 (5)	1.400 (0.598)
	2.1.4 Navigation	0 (0)	3 (15)	10 (50)	7 (35)	2.200 (0.696)
	2.1.5 Location in the website	0 (0)	0 (0)	9 (45)	11 (55)	2.550 (0.510)
	2.1.6 Color scheme	0 (0)	10 (50)	6 (30)	4 (20)	1.700 (0.801)
**2.2 Consistency, n (%)**						
	2.2.1 Page layout	0 (0)	0 (0)	13 (65)	7 (35)	2.350 (0.489)
	2.2.2 Navigation links	0 (0)	0 (0)	20 (100)	0 (0)	2.000 (0.000)
	2.2.3 Website structure	0 (0)	0 (0)	20 (100)	0 (0)	2.000 (0.000)
**2.3 Functionality, n (%)**						
	2.3.1 Search facility	0 (0)	7 (35)	13 (65)	0 (0)	1.650 (0.489)
	2.3.2 Browsing facility	0 (0)	5 (25)	11 (55)	4 (20)	1.950 (0.686)
	2.3.3 Cognitive overhead	0 (0)	0 (0)	20 (100)	0 (0)	2.000 (0.000)
	2.3.4 Navigation tools	0 (0)	0 (0)	20 (100)	0 (0)	2.000 (0.000)
	2.3.5 Third-party plug-ins	0 (0)	0 (0)	20 (100)	0 (0)	3.000 (0.000)
**2.4 Engageability, n (%)**						
	2.4.1 Effective judgement	0 (0)	0 (0)	20 (100)	0 (0)	2.000 (0.000)
	2.4.2 Interactivity	0 (0)	3 (15)	17 (85)	0 (0)	1.850 (0.366)
	2.4.3 Personalized experience	0 (0)	2 (10)	14 (70)	4 (20)	2.100 (0.553)
	2.4.4 Integration of nontextual media	0 (0)	12 (60)	8 (40)	0 (0)	1.400 (0.503)
**3.1 Currency, n (%)**						
	3.1.1 Recent events	0 (0)	3 (15)	17 (85)	0 (0)	1.850 (0.366)
	3.1.2 Submit comments	0 (0)	14 (70)	6 (30)	0 (0)	1.600 (0.940)
	3.1.3 Updated	1 (5)	1 (5)	1 (5)	17 (85)	2.700 (0.801)
**3.2 Conflicts of interest, n (%)**						
	3.2.1 Who runs the website?	0 (0)	0 (0)	0 (0)	20 (100)	3.000 (0.000)
	3.2.2 Pay for the website	0 (0)	2 (10)	18 (90)	0 (0)	1.900 (0.308)
	3.2.3 Declaration of objectives	0 (0)	5 (25)	4 (20)	11 (55)	2.300 (0.865)
**3.3 Content production, n (%)**						
	3.3.1 Content production method	0 (0)	1 (5)	19 (95)	0 (0)	1.950 (0.224)
	3.3.2 Robust method	0 (0)	9 (45)	11 (55)	0 (0)	1.550 (0.510)
	3.3.3 Original sources	0 (0)	0 (0)	20 (100)	0 (0)	2.000 (0.000)

### Quality of Breast Cancer Papers

#### Value

The evaluation of the value of the papers was mainly based on seven questions. The questions were used to evaluate the treatment (or treatments) described in the publication. The overall rating of the value of the papers on breast cancer was low. The highest scores were for items on whether the description indicated that there may be more than one possible treatment choice (item 14) and on whether the paper supports shared decision making (item 15). The items with low scores included information about the risks of each treatment (item 11), what would happen without treatment (item 12), and how the treatment choices affect the overall quality of life (item 13). The specific results are shown in Table 3.

**Table 3:** Descriptive statistics of the DISCERN items.

Item	Score=1, n (%)	Score=2, n (%)	Score=3, n (%)	Score=4, n (%)	Score=5, n (%)	Mean (SD)
#9. Does it describe how each treatment works?	16 (27)	20 (34)	14 (23)	8 (13)	2 (3)	2.333 (1.115)
#10. Does it describe the benefits of each treatment?	7 (11)	16 (27)	28 (47)	6 (10)	3 (5)	2.700 (0.979)
#11. Does it describe the risks of each treatment?	24 (40)	18 (30)	12(20)	4 (7)	2 (3)	2.033 (1.089)
#12. Does it describe what would happen if no treatment were used?	44 (73)	11 (18)	3 (5)	1 (2)	1 (2)	1.400 (0.807)
#13. Does it describe how the treatment choices affect overall quality of life?	25 (42)	16 (27)	12(20)	4 (6)	3 (5)	2.067 (1.163)
#14. Is it clear that there may be more than one possible treatment choice?	2 (3)	15 (25)	9 (15)	9 (15)	25 (42)	3.667 (1.336)
#15. Does it provide support for shared decision making?	3 (5)	14 (23)	14 (23)	13 (22)	16 (27)	3.417 (1.253)

#### Suitability

The suitability evaluation results of the papers are shown in Table 4. In this study, after evaluation, the highest SAM score was 42 points (100%). Of 60 papers, only 1 (1.67%) met the criteria for superior suitability, as established by SAM, 27 (45%) papers met the criteria for adequate suitability, and 32 (53.33%) papers were evaluated as not suitable material. The graphics (0.85/8 points), literacy demand (4.18/10 points), and layout and typography (2.53/6 points) scores were low, and the graphics score was the lowest. Three other aspects were also evaluated: content (3.82/6 points), cultural appropriateness (1.85/4 points), and learning stimulation (2.63/6 points).

**Table 4:** Descriptive statistics of SAM^a^ items.

Factor		Score=0^b^	Score=1^c^	Score=2^d^	Mean (SD)
**Content, n (%)**					
	1) It is important that readers understand the purpose of the materials. If they do not, they may miss the main point.	4 (6)	28 (47)	28 (47)	1.400 (0.616)
	2) Adult learners usually want to solve their problems rather than learn facts. The content of most interest and use is likely to be behavior information to help solve their problems.	1 (2)	57 (95)	2 (3)	1.017 (0.225)
	3) Scope should be limited to the purpose/objectives of the material and to what can reasonably be learned in the time typically allocated to reading the information.	11 (18)	39 (65)	10 (17)	0.983 (0.596)
	4) A summary offers readers a chance to see the key points in other words or examples. They are important; readers often miss the key points when they first read them.	36 (60)	23 (38)	1 (2)	0.417 (0.530)
**Literacy demand, n (%)**					
	1) The text reading level is an important factor in whether your target group understands your document.	26 (43)	32 (54)	2 (3)	0.600 (0.558)
	2) A conversational style and active voice lead to easy-to understand text. Simple sentences are used extensively.	3 (22)	46 (76)	1 (2)	0.800 (0.443)
	3) It is best to use common, explicit words and avoid words that express general terms.	0 (0)	59 (98)	1 (2)	1.017 (0.129)
	4) We learn new facts/behaviors more quickly when told the context first.	16 (27)	43 (71)	1 (2)	0.750 (0.474)
	5) Headers or topic captions tell briefly what is coming up next. These “road signs” make the text look less formidable, and prepare the reader’s thought process to expect the next topic.	7 (12)	45 (75)	8 (13)	1.017 (0.504)
**Graphics, n (%)**					
	1) Simple line drawings can promote realism without including distracting details. Visuals are accepted and remembered better when they portray what is familiar and easily recognized.	44 (73)	14 (24)	2 (3)	0.300 (0.530)
	2) Non-essential details, such as room background, elaborate borders, and unneeded color, can distract the reader, whose eyes may be “captured” by these details. The illustrations should visually represent the key points.	44 (73)	12 (20)	4 (7)	0.333 (0.601)
	3) Many readers do not understand the purpose of lists, charts, and graphs. Explanations and directions are essential.	55 (92)	2 (3)	3 (5)	0.133 (0.468)
	4) Captions can quickly tell the reader what the graphic is all about and where to focus within the graphic. A graphic without a caption is usually an inferior instruction and a missed learning opportunity.	55 (92)	5 (8)	0 (0)	0.083 (0.279)
**Layout and typography, n (%)**					
	1) Layout has a substantial influence on the suitability of materials.	23 (38)	34 (57)	3 (5)	0.667 (0.572)
	2) Type size and fonts can make text easy or difficult for readers at all skill levels.	0 (0)	60 (100)	0 (0)	1.000 (0.000)
	3) Few people can remember more than seven independent items. For adults with low literacy skills, the limit may be three- to five-item lists. Longer lists need to be broken into smaller chunks.	17 (28)	34 (57)	9 (15)	0.867 (0.650)
**Learning stimulation and motivation, n (%)**					
	1) When a reader responds to an instruction, chemical changes take place in the brain that enhance retention in long-term memory. Readers should be asked to solve problems, to make choices, to demonstrate, etc.	36 (60)	19 (32)	5 (8)	0.483 (0.651)
	2) People often learn more readily by observation, by doing something for themselves rather than by reading or being told, and when specific, familiar instances are used rather than the abstract or general.	8 (13)	36 (60)	16 (27)	1.133 (0.632)
	3) People are more motivated to learn when they believe the tasks/behaviors are doable by them.	2 (3)	55 (92)	3 (5)	1.017 (0.291)
**Cultural appropriateness, n (%)**					
	1) A valid measure of cultural appropriateness of material is how well its logic, language, and experience (inherent in the instruction) match the logic, language, and experience of the intended audience.	12 (20)	41 (68)	7 (12)	0.917 (0.561)
	2) To be accepted, an instruction must present cultural images and examples in realistic and positive ways.	6 (10)	52 (87)	2 (3)	0.933 (0.362)

^a^SAM: Suitability Assessment of Materials.

^b^Score 0: not suitable.

^c^Score 1: adequate.

^d^Score 2: superior.

#### Accuracy

For originality and for listing references, the scores were generally low. Of the 60 papers, 44 (73%) were unoriginal and 46 (77%) did not have a bibliography or resource list available. The top three items were “whether the paper contains errors or omissions”, “whether the paper is misleading or biased,” and “whether the paper is accurate.” More than 97% (n=58) of the papers were correct in their content descriptions, with no errors, omissions, or misleading hints. The evaluation results are shown in Table 5.

**Table 5:** Descriptive statistics of the Michigan Checklist items.

Item	Score=–2, n (%)	Score=+2, n (%)	Score=–3, n (%)	Score=+3, n (%)
#18. Are sources cited or credited?	—^a^	—	44 (73)	16 (27)
#19. Is a bibliography or resource list available?	46 (77)	14 (23)	—	—
#20. Can you identify errors or significant omissions in information presented?	—	—	2 (3)	58 (97)
#21. Are opinions or misleading/biased information presented as fact?			1 (2)	59 (98)
#22. Does information presented as factual appear to be accurate to the best of your knowledge?	1 (2)	59 (98)	—	—
#23. Is there an identifiable conflict of interest?	14 (23)	46 (77)	—	—

^a^N/A: not applicable.

## Discussion

### Principal Findings

Generally, the quality of Chinese breast cancer websites is poor. The quality of the online papers on breast cancer is also poor. The quality of online information service platforms, which are an important medium for the dissemination of health information in the new media environment [34], affects the public's health decisions [35].

Similar to the results of previous research, this study found that the format (updated and who runs the website) of Chinese breast cancer websites is good [36], but the color scheme, text setting, function of user comment submission, and language design should be improved. For example, using colors to mark the title can make the paper clearer, but this setting is not effective for people with color cognitive impairment. Another similar issue is font size; rather than fancy colors, older users want to be able to read information with a larger font size and higher contrast than younger users. For user groups such as the elderly, special services and personalized layout options can be provided, which requires further thinking by health website owners. Another prominent problem is that the user comment submission function is poor. Pang’s study [37] showed that health websites should provide functions for story sharing and memorials for women. These functions provide an outlet for women to share their feelings of grief and loss [37]. Therefore, website designers should focus on personalization and provide a comment section to allow users to submit comments and share experiences on specific content. In addition, China is a multiethnic country, and many ethnic groups have their own languages. Given that health websites are for users nationwide, almost all the websites in this study fail to support multiple languages, which greatly limits the effective dissemination of health information on the websites.

The quality of online breast cancer treatment information is poor. Online treatment information is of little value to users making breast cancer treatment decisions. Although the evaluation of the value of treatment options presents a “modest” result, the assessment of the benefits and risks of treatment is low, and online papers on breast cancer treatment tend to give compromised and biased advice. Although patients with breast cancer have a clear and urgent need for treatment information [38], the results of this study suggest that doctors are still the most valuable source of information for patients who want to know more about breast cancer treatment. Therefore, breast cancer website developers should provide easy-to-understand online health information that meets the needs of breast cancer patients and is useful for treatment.

Regarding the suitability of papers on breast cancer treatment, most of the papers have easy-to-understand titles that clearly describe the purpose of the papers. The layout and cultural appropriateness are also good. However, regarding the use of pictures, some papers use pictures with little relevance and that lack explanatory descriptions. Tables are rarely used, and table captions are lacking. Especially with regard to some professional medical knowledge, the lack of descriptions often makes it difficult for users to understand the desired health information on a deeper level. Another important problem we found was that many websites do not give proper explanations of medical terms, which increases the users’ difficulty in reading and increases the level of literacy required to understand the text. Certainly, a few health websites in China have realized this problem, such as 39 Health Network^TM^ [39], which provides hyperlinks to detailed explanations of medical terms to help users better understand the health information disseminated. However, most websites fail to do this. Therefore, to better enable users to understand the online health information they seek, health websites should cooperate with professional doctors, nurses, and health care providers, making full use of the professional advantages of medical personnel and providing effective guidance to internet users consuming the online health information.

Although the results of the manual evaluation of the accuracy of the papers by tumor surgeons indicated that the papers are not obviously wrong, biased, or misleading, most papers quoted others (44/60, 73%) and did not provide references or a resource list (46/60, 77%). We randomly selected eight papers and tried to search them using the Baidu search engine. The results showed that these eight papers exist on a large number of websites at the same time. For papers, indifference to copyright is an urgent problem in the dissemination of online health information in China that must be solved. Website operators should strengthen the copyright awareness of online information and regulate their own information publishing behavior. The problem of protecting the copyright of online health information may also be an important research topic in the future.

In addition, there are extensive recessive advertisements (the headline or image often contains attractive health-related information, but when you click the link, it turns out to be a page designed to sell products) on all the large health websites, and they are usually embedded in a page as a picture or video. We assessed the accuracy of health knowledge disseminated in several papers that contained recessive advertisements and found no significant errors. However, the existence of such recessive advertisements still leads users to have negative subjective feelings, reduces trust in the content of the papers, and may mislead users with regard to their health behaviors. Once misled by such information, users may choose less mature treatment methods, even leading to the delay of standard treatment [40]. In this regard, we are reminded of the Wei Zexi incident, which was a tragic case of a young man who died because he trusted false medical information on the internet and chose inappropriate treatment for a disease [41]. Although the *Technical Manual for the Generation and Dissemination of Health Science Information (Media Edition)* and *Recommendations for Public Recognition and Utilization of Online Health Information (2017 Edition)* were published in 2017, monitoring the reliability and accuracy of online health information remains an important task in China [42]. The Chinese government, however, needs to strengthen the monitoring of the quality of online health information to prevent such information from misleading the public with regard to diagnosis and treatment behavior.

### Limitations

This study had some limitations. First, we chose influential websites as the research objects of this paper according to their traffic rankings and excluded some websites with low traffic, which caused selection bias. Second, although there are some assessment tools for the readability of written materials (eg, SMOG), to the best of our knowledge, there is no assessment tool for the readability of Chinese written materials. Therefore, an evaluation of the readability of written materials in this study was lacking. Third, video and animation are more important than textual information for users to understand health information. However, due to the lack of relevant evaluation tools, we could not evaluate the quality of these types of multimedia information. Finally, all evaluations were performed by researchers, and their perceptions may differ from those of users. Despite these limitations, the results of this study are still highly valuable for improving the quality of Chinese online health information about breast cancer.

### Conclusion

In this study, we found that the quality of Chinese breast cancer websites is poor and that the quality of online health information is not ideal. Most websites can provide users with a convenient and easy-to-use breast cancer information retrieval platform, but the breast cancer–related health information they publish is of little value for users making decisions about breast cancer treatment. At the same time, there are also some problems, such as difficulty in tracing the source of information, a lack of copyright awareness, and a lack of advertising supervision. Therefore, developers should design health websites that meet the needs of breast cancer users [43]. Breast cancer users should choose trustworthy health websites that provide accurate information. The government should strengthen the standardized management of health websites to ensure that the health information published on the websites is accurate, up to date, and effective. In addition, the government should strengthen cooperation between websites and medical professional organizations, such as by establishing professional medical customer services and official WeChat accounts, to ensure that users can obtain effective guidance and suggestions from medical professionals when using online health information.

## Appendix

Multimedia Appendix 1 Searching and screening flow for breast cancer websites and papers.

## Abbreviations

BBMC: BengBu Medical College
HONcode: Health on the Net Foundation Code of Conduct
ICP: internet content provider
SAM: Suitability Assessment of Materials
SMOG: Simplified Measure of Gobbledygook
SPSS: Statistical Package for Social Sciences
URL: Uniform Resource Locator
