# Supplementary figures and images for: Assessing the Quality of Online Health Information About Breast Cancer from Chinese Language Websites: Quality Assessment Survey
Source: JMIR Cancer. 2021 Nov 18;7(4):e25783. doi: 10.2196/25783 (PMC8663447; doi:10.2196/25783)

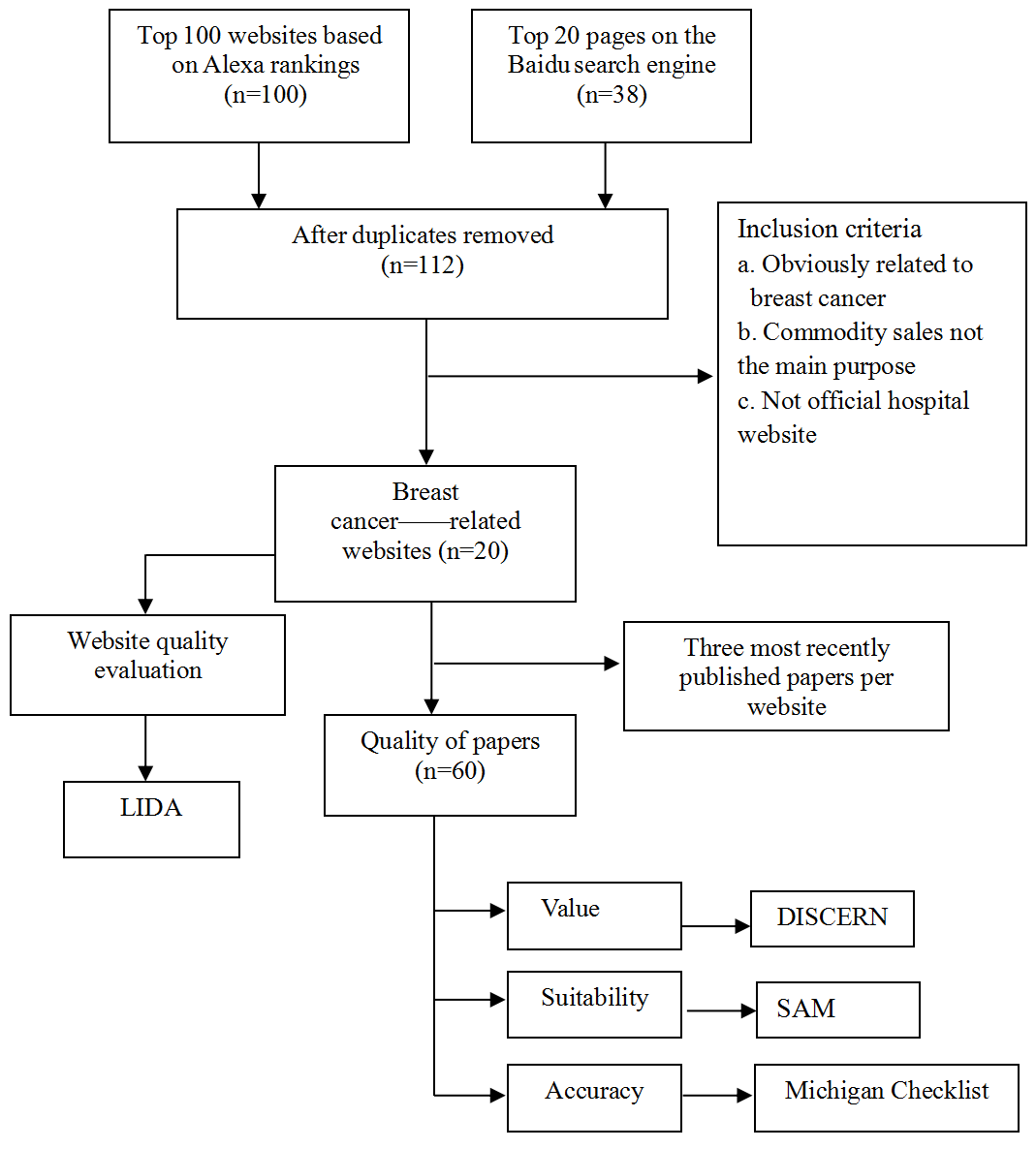

Supplement: Multimedia Appendix 1 [file cancer_v7i4e25783_app1.png]
